# Body image during pregnancy: an evaluation of the suitability of the body attitudes questionnaire

**DOI:** 10.1186/1471-2393-12-91

**Published:** 2012-09-06

**Authors:** Matthew Fuller-Tyszkiewicz, Helen Skouteris, Brittany Watson, Briony Hill

**Affiliations:** 1 School of Psychology, Deakin University, 221 Burwood Highway, Burwood, Victoria, 3125, Australia

**Keywords:** Pregnancy, Body dissatisfaction, Body attitudes questionnaire, Measurement invariance

## Abstract

**Background:**

Available data suggest that body dissatisfaction is common during pregnancy and may even be a precursor to post-natal depression. However, in order to accurately identify at-risk women, it is essential to first establish that body image measures function appropriately in pregnant populations. Our study examines the suitability of the Body Attitudes Questionnaire (BAQ) for measuring body dissatisfaction among pregnant women by comparing the psychometric functioning of the BAQ: (1) across key phases of pregnancy, and (2) between pregnant and non-pregnant women.

**Methods:**

A total of 176 pregnant women from Melbourne, Victoria filled out a questionnaire battery containing demographic questions and the Body Attitudes Questionnaire at 16, 24, and 32 weeks during pregnancy. A comparison group of 148 non-pregnant women also completed the questionnaire battery at Time 1. Evaluations of the psychometric properties of the BAQ consisted of a series of measurement invariance tests conducted within a structural equation modelling framework.

**Results:**

Although the internal consistency and factorial validity of the subscales of the BAQ were established across time and also in comparisons between pregnant and non-pregnant women, measurement invariance tests showed non-invariant item intercepts across pregnancy and also in comparison with the non-pregnant subgroup. Inspection of modification indices revealed a complex, non-uniform pattern of differences in item intercepts across groups.

**Conclusions:**

Collectively, our findings suggest that comparisons of body dissatisfaction between pregnant and non-pregnant women (at least based on the BAQ) are likely to be conflated by differential measurement biases that serve to undermine attempts to accurately assess level of body dissatisfaction. Researchers should be cautious in assessments of body dissatisfaction among pregnant women until a suitable measure has been established for use in this population. Given the fact that body dissatisfaction is often associated with maladaptive behaviours, such as unhealthy eating and extreme weight loss behaviours, and with ante-and post-natal depression, that have serious negative implications for women’s health and well-being, and potentially also for the unborn foetus during pregnancy, developing a suitable body image screening tool, specific to the perinatal period is clearly warranted.

## Background

Body image is a broad term used to capture the cognitive, affective, behavioural, and perceptual aspects of one’s experience of her/his body [1]. Body dissatisfaction is one facet of body image relating to the degree of dissatisfaction with particular aspects of the body [2]. Body dissatisfaction is common in the general population [3], and appears to be more prevalent among women than men [4,5]. Prominent theories, such as Objectification Theory [6] and Tripartite Influence Model [7], argue that appearance-related socio-cultural values foster body image disturbances by strongly promoting an idealized physique – thin and toned for women and a lean, muscular shape for men – that departs markedly from the average physique, and which is unrealistic for most individuals to attain [8].

These models of body dissatisfaction derive largely from samples of females aged 18–25 [8], although there is increasing sampling of early and pre-adolescent cohorts in order to confirm the purported origins of body image issues [9,10]. However, studies of other subgroups in which the human body undergoes considerable change, such as pregnant women, may provide further insights into the development and maintenance of body dissatisfaction. Pregnancy is characterised by significant physiognomic and psychosocial changes, such as hormonal fluctuations, the experience of pregnancy-related physical symptoms and changes to one’s appearance (e.g., rapid weight gain, nausea, back ache, varicose veins, stretch marks, acne, and swollen ankles and feet), and changing relationship dynamics with partner, family, and friends [11]. Given that during pregnancy a woman’s body increases in size, her body shape changes, and pregnancy-related physical symptoms become more pronounced, women who retain societal standards of appearance are likely to experience increased body dissatisfaction. The extent to which pregnant women are able to reject the thin ideal and/or adopt more realistic appearance-related values during pregnancy may explain maintenance or reduction in body dissatisfaction. Hence, compared to other times in women’s lives when body shape remains relatively stable, pregnancy may allow for a more powerful test of the factors leading to body dissatisfaction [11].

Although accumulated research findings clearly suggest that body image concerns are prevalent in pregnant women [12-14], findings have been mixed regarding whether the severity of such concerns are equivalent to or greater than in non-pregnant cohorts [9]. One cause of these mixed results is over-reliance on general samples of pregnant women, without due consideration of how body image concerns may change across the phases of pregnancy. There is a surfeit of cross-sectional studies, making it difficult to delineate substantive differences in body image disturbances across pregnancy from sample-specific differences and sampling error. Findings from the few longitudinal studies that have tracked body image issues across pregnancy suggest that body image concerns may peak in early pregnancy and again in post-partum, and that there may be a period of relative satisfaction during mid to late pregnancy [12,13,15,16]. It is also evident that the salience of body shape and size is heightened in early pregnancy relative to late pregnancy [12,13] and women feel stronger, fitter, and less fat later in pregnancy compared with early pregnancy [12,13,16].

The veracity of these and similar findings may also be undermined by reliance upon body image scales that have been validated for use in non-pregnant rather than pregnant populations [11]. Any potential group difference (or indeed failure to find a group difference) in body dissatisfaction may be attributed to one or more of the following sources: (1) measurement error; (2) response style differences across cohorts; (3) qualitative differences in the meaning of the underlying construct; or (4) substantive differences in the construct. While researchers often assume that differences are of a substantive nature (particularly when internal consistency, test-retest, and predictive validity estimates are satisfactory), without further testing we cannot rule out the influence of measurement biases (i.e., reasons 1–3) [17].

One method to investigate this issue is the statistical technique of measurement invariance. This can be used to disambiguate the joint effects of measurement biases and substantive differences that are evident between different populations who use the same measure [18]. This approach evaluates the presence of four common forms of measurement bias: (1) factor structure (does the scale have the same number of underlying factors across groups?); (2) factor loadings (does the scale convey the same meaning across groups?); (3) item intercepts (do groups differ in their response profiles, for instance, does one group exhibit a more acquiescent response style?), and (4) item residual variances (is item true score measurement more reliable in one group than another?). Unless it has been demonstrated that the scale is free of any of these forms of measurement bias, one may question the validity of conclusions about substantive group differences [18].

### Aims and rationale

Despite the importance of ensuring measurement equivalence before testing for group differences in a given construct, the suitability of available measures of body dissatisfaction for use in pregnant populations has yet to be evaluated empirically. Therefore, the present study used the Body Attitudes Questionnaire [19] to address the following two key research questions:

1) Does the scale function equivalently across three time points in pregnancy?

2) Does the scale function equivalently for pregnant and non-pregnant women?

The BAQ is one of the most commonly used measures of body dissatisfaction among pregnant women because it comprises four subscales of dissatisfaction that are, at face value, relevant for this population: feeling fat, strength and fitness, salience of weight and shape, and attractiveness [11]. However, in light of the noted physiognomic and psychological changes that manifest in pregnancy, it is likely that the constructs measured by the BAQ will take on a different meaning across the various phases of pregnancy. Therefore, it is predicted that the BAQ will exhibit non-invariance across pregnancy and also in comparisons between pregnant and non-pregnant women.

## Method

### Participants

The present sample consisted of 324 women (148 non-pregnant and 176 pregnant women). Pregnant women (*M* = 30.77 years, *SD* = 4.31, range = 18–41 years) were significantly older than non-pregnant women (*M* = 27.06 years, *SD* = 6.24, range = 18–40 years); *t*_(df=322)_ = 6.30, *p* < .001, Cohen’s *d* =0.70. The majority of non-pregnant women were born in Australia (83.3%); similarly, 85.1% of pregnant women were born in Australia. Non-pregnant women had, on average, a lower Body Mass Index (BMI, *M* = 24.59, *SD* = 4.89) than pregnant women in the early stages of the second trimester (*M* = 27.10, *SD* = 5.68); *t*_(df=322)_ = 4.22, *p* < .001, Cohen’s *d* =0.47, which was Time 1 of assessment. Pregnant women significantly increased their BMI from Time 1 (T1, *M =* 16.66 weeks gestation*, SD* = .89 weeks) to Time 2 (T2, *M =* 24.60 weeks gestation*, SD* = .80 weeks) (BMI_T2_ = 28.75, *SD* = 5.76, *t*_(df=350)_ = 2.71, *p* < .01, *d* = 0.29), and from T2 to Time 3 (T3, *M =* 32.97 weeks gestation*, SD =* .85 weeks) (BMI_T3_ = 30.02, SD = 5.92, t _(df=350)_ = 2.04, *p* < .05, *d* = 0.22). Although pregnant women were more likely to have exercised in the past month (89.6% versus 70%; χ_(df =1)_^2^ = 19.76, *p* < .001, Cramer’s V = .17), the amount and type of exercise they engaged in were less extreme (see Table 1).

**Table 1:** Breakdown of demographic differences across groups

	**Pregnant women (*n* = 176)**	**Non-pregnant women (*n*= 148)**	**χ**^**2**^**or t**^**^**^
Exercise (min/week)	128.50 (91.59)	192.18 (136.05)	5.01**
Exercise intensity			
Low	66.8%	31.8%	40.06***
Moderate	30.2%	42.4%	5.43*
Vigorous	3.0%	25.8%	36.42***
			
Number of babies^#^			
0	53.3%	62.7%	2.52
1	35.6%	16.0%	22.45***
2	6.1%	16.7%	16.92***
3	3.9%	4.6%	8.49**
4+	1.1%	0.0%	0.84
			
Mental illness			
Minor depression	17.0%	24.0%	2.63
Major depression	5.5%	6.0%	0.03
Antenatal depression	1.1%	2.0%	0.42
Postnatal depression	4.9%	10.0%	2.96
Bipolar disorder	1.1%	2.0%	0.42
Anxiety disorder	15.4%	14.7%	0.01
Eating disorder	1.1%	8.0%	9.45**
Substance/alcohol abuse	1.1%	1.3%	0.03

^^^Chi square for comparisons of frequencies, t values for comparisons of mean scores.

^#^Not including current pregnancy.

The majority of the sample was university educated; 37.3% of non-pregnant women and 42.6% of pregnant women had a bachelor’s degree, while a further 23.3% of non-pregnant women and 16.6% pregnant women had postgraduate qualifications. Furthermore, most of the non-pregnant women and pregnant women during the first trimester were employed (76% versus 76.8%).

There was a significant difference in the relationship status of non-pregnant and pregnant women in the present study; χ_(df =4)_^2^ = 72.17, *p* < .001, Cramer’s V = .33. Seventy-five percent of pregnant women were married, 22.5% were in a de facto relationship, and 2.2% were never married/single. In contrast, 40% of the non-pregnant women were married, 34.7% were never married/single, 20% were in a de facto relationship, 3.3% were separated from their spouse, and 2% were widowed or divorced. Likewise, there was a difference in number of children (excluding current pregnancy for pregnant cohort); χ_(df =4)_^2^ = 22.45, *p* < .001, Cramer’s V = .19. As shown in Table 1, pregnant women were more likely than non-pregnant women to have multiple children (specifically, 1 or 2 children).

The non-pregnant cohort were more likely to have a history of mental illness (46.7% versus 35.2%); χ_(df =1)_^2^ = 5.68, *p* < .05, Cramer’s V = .09. However, the only psychological condition to differentiate between the two groups was history of eating disorders (see Table 1).

### Measures

Demographic questions were used to assess participants’ age, place of birth, relationship status (married/single/de facto, etc.), number of children, employment status, education, exercise habits, history of mental illness, and height and weight (to calculate BMI).

The following four subscales from the Body Attitudes Questionnaire [19] that are most suitable for pregnant women were used to assess self-perceived appearance and bodily function: (1) feeling fat (e.g., ‘I feel fat when I can’t get clothes over my hips’); (2) strength and fitness (‘I quickly get exhausted if I overdo it’); (3) salience of weight and shape (‘I spend a lot of time thinking about my weight’), and (4) attractiveness (‘People hardly ever find me sexually attractive’). Items were rated on a 5-point Likert-type scale ranging from 1 (*definitely disagree*) to 5 (*definitely agree*). Items were scored so that higher scores reflect greater attractiveness, feeling fat, salience of appearance, and strength/fitness.

Ben-Tovim and Walker demonstrated the factorial validity and internal consistency (α = .87 for full scale) of the BAQ, and showed that scores on these subscales were stable over a four-week test-retest period (*r* = .64 for salience of weight and shape to *r* = .91 for feeling fat) in a non-clinical sample of hospital employees and students [19]. Similarly, Skouteris and colleagues demonstrated internal consistency and stability in BAQ subscales across three time points during pregnancy: Time 1 (16–23 weeks), Time 2 (24–31 weeks) and Time 3 (32–39 weeks). The internal consistency estimates (averaged over the three time points) ranged from .70 (strength and fitness) to .88 (feeling fat), whereas the averaged test-retest reliability estimates ranged from .64 (salience of weight and shape) to .77 (feeling fat) [16].

In the present study, reliability estimates were acceptable for both pregnant and non-pregnant participants. For non-pregnant women, Cronbach’s alpha values were .92 (feeling fat), .80 (attractiveness), .84 (salience), and .81 (strength/fitness). Internal consistency estimates were slightly lower for pregnant women: .62 - .69 (attractiveness), .91 - .93 (feeling fat), .75 - .81 (salience), and .76 - .79 (strength/fitness). However, scores on these subscales were quite stable across the three time points for pregnant women: .66 - .80 (attractiveness), .69 - .77 (feeling fat), .65 - .73 (salience), and .67 - .74 (strength/fitness).

### Procedure

Permission to undertake the study was obtained from the Deakin University Ethics Committee. Pregnant participants were primarily recruited through advertisements in parenting magazines and general media advertising; however, mother, child, and baby forums and obstetrician clinics were also targeted. To recruit the non-pregnant subsample, we used social media sites and general media advertising. These advertisements invited women to participate in a study examining body image in women; advertisements targeting pregnant women specified that we were interested in tracking body image among pregnant women across three time points of pregnancy. Women who registered interest in the study were mailed a hard copy of the questionnaire with a reply paid envelope (T1). Pregnant women were recruited at or after 16 weeks gestation. This standardised time period allowed for women to learn about their pregnancy, consider the pregnancy certain (since the threat of miscarriage has subsided) and to then participate at regular 8-week time points.

The same questionnaire (minus questions about relationship status, number of children, exercise habits, history of mental illness, country of birth, and educational attainment) was mailed again to pregnant women 8 weeks later at approximately 24 weeks (T2) and again 8 weeks later at approximately 32 weeks (T3) of their pregnancy. Questionnaires for pregnant women were coded in order to link data across the three waves of data collection for each woman.

### Data analytic strategy

Data were analysed in Mplus 6.1, using robust (mean- and variance-adjusted) maximum likelihood estimation (MLMV) for continuous indicator variables. These estimators are robust to issues of non-normality [20]. Missingness (less than 5% overall) was handled using maximum likelihood estimation under the assumption that data were missing at random (MAR)[21].

Given that comparisons of BAQ subscales across the stages of pregnancy constitutes a repeated measures design, a single augmented means and covariance matrix approach was undertaken in which items were correlated across time to control for non-independence of scores from T1 to T3. In the event that full measurement invariance was established for these three time points, non-pregnant women were to be compared against T1 (*M =* 16.66 weeks gestation, *SD* = .89) data since this was the only time point without missing data and, therefore, would not require imputation for missing values. However, as we were unable to establish measurement invariance across time (see below), non-pregnant women were instead compared to each separate time point to see if measurement invariance could be established between non-pregnant and pregnant women at any of the stages of pregnancy. Comparisons between pregnant and non-pregnant women were conducted using multi-group confirmatory factor analysis.

Adequacy of baseline model fit (i.e., the factor structure without imposition of cross-temporal or cross-group equality constraints on parameters) was examined using the following criteria: Comparative Fit Index (CFI > .95 for good fit, > .90 for adequate fit), Root Mean Square Error of Approximation (RMSEA; RMSEA ≤ .06 for good fit, RMSEA < .08 for adequate fit), and Standardized Root Mean Square Residual (SRMR; SRMR < .05 for good fit, SRMR < .08 for adequate fit) [22,23].

Once adequate model fit was established for each group or time point separately, four increasingly stringent invariance assumptions were tested in sequence, starting with the least restrictive model. The first model (configural invariance) required that items loaded onto the same factors across data sets, but allowed item parameters (factor loadings, residual variances, and intercepts), factor variances, and latent means to vary across groups or time. In the second model (weak invariance), equality constraints across groups (or time points) were applied to factor loadings and model fit was re-evaluated. Evidence of adequate fit for this model ensures that a given factor has the same meaning across groups (or time) [18]. Strong invariance (model three) involved constraining item intercepts to equality across groups (or time) to evaluate potential for systematic bias in responses from one group to another (or from one time point to another). If the assumptions of strong invariance held, then an additional equality constraint was to be placed on residual variances (model four – strict invariance). This last step ensures that group (or time-related) differences obtained from comparisons of item composite scores (i.e., summing and averaging across individual items) can be attributed to substantive differences on the construct and are not due to differences in proportion of error variance in item-level scores.

Measurement invariance is statistically evaluated by calculating differences in fit indices (typically, Δχ^2^, ΔCFI, etc.) between reference and comparison models. The target model is typically compared against a less restrictive model (e.g., comparing model one versus the baseline model). As χ^2^ is sensitive to sample size and also to minor departures from normality [24,25], some researchers advocate the use of practical changes in model fit, using one of several comparative fit measures (e.g., CFI or TLI) [26]. The present study used ΔCFI > .01 to indicate practical change in fit from one model to the next, as recommended by Cheung and Rensvold [26]. Model comparison terminated if equality constraints led to practical change in CFI values.

To the extent that one of the proposed models did not adequately fit the data, the researchers examined modification indices to determine sources of variance across groups and freed the equality constraint for that particular parameter. If this revised model is shown to have adequate model fit, it may be concluded that the measure exhibits *partial* invariance [18,27].

## Results

### Tests of invariance across pregnancy

As shown in Table 2, each of the BAQ subscales (with the exception of the feeling fat subscale) was adequately represented by a uni-dimensional model. Examination of the modification indices suggested that the feeling fat subscale could be divided into two related subscales for our pregnant subgroup: (1) feeling fat – general (items 4, 8, 10, 25, 28, 35, and 38; numbers aligned with those reported in Ben-Tovim & Walker [19]), and (2) feeling fat – clothing specific (items 5, 14, 19, 42, and 44). As the two factor model of the feeling fat subscale also provided a significantly better fit than the uni-dimensional model for non-pregnant women (as covered in the next subsection), subsequent measurement invariance tests of the feeling fat subscale separated the items into these two identified subcomponents.

**Table 2:** Tests of measurement invariance across time for pregnant cohort (*n* = 176)

							**90% CI**	
**Subscale**	**χ**^**2**^	**df**	**CFI**	**ΔCFI**	**SRMR**	**RMSEA**	**LOW**	**HIGH**
*Feeling fat*								
Configural	1084.346	555	.884	-	.063	.074	.067	.080
*Feeling fat_revf1*								
Configural	285.266	165	.945	-	.057	.064	.052	.077
Weak	310.608	177	.939	.006	.065	.065	.053	.077
Strong	353.572	191	.925	.014	.067	.070	.058	.081
*Feeling fat_revf2*								
Configural	177.214	72	.927	-	.059	.091	.074	.108
Weak	190.807	80	.923	.004	.064	.089	.073	.105
Strong	226.498	90	.905	.018	.068	.093	.078	.108
*Attractiveness*								
Configural	112.800	72	.957	-	.057	.057	.035	.076
Weak	125.437	80	.952	.005	.070	.057	.037	.075
Strong	198.437	90	.885	.067	.072	.083	.067	.098
*Weight/shape salience*								
Configural	157.946	72	.934	-	.059	.082	.065	.100
Weak	165.050	80	.935	.001	.062	.078	.061	.095
Strong	199.908	90	.916	.019	.064	.083	.068	.099
*Strength & fitness*								
Configural	223.646	114	.922	-	.063	.074	.059	.088
Weak	240.331	124	.917	.005	.072	.073	.059	.087
Strong	279.244	136	.898	.019	.075	.077	.064	.090

Notes: Feeling fat_revf1 and revf2 represent the feeling fat-general and feeling-fat clothing specific factors.

The imposition of equality constraints on factor loadings over time produced minimal change in CFI values (all ΔCFIs < .01), suggesting that the subscales retained the same meaning across the three phases of pregnancy. However, additional equality constraints on item intercepts led to non-ignorable changes in CFI values: ΔCFI = .014 for feeling fat-general, ΔCFI = .018 for feeling fat – clothing specific, ΔCFI = .067 for attractiveness, ΔCFI = .019 for salience, and ΔCFI = .019 for strength and fitness. Cross-temporal invariance tests were terminated at this step. Differences in item intercepts across time are presented in Additional file 1.

### Comparison between pregnant and non-pregnant women

As the subscales of BAQ were non-invariant across the phases of pregnancy, comparisons with non-pregnant women were made at each time point separately. However, it was important to first ensure that factor structure suggested by Ben-Tovim and Walker [19] could be replicated in our non-pregnant sample. As shown in Table 3, each of the subscales was adequately represented by uni-dimensional models. RMSEA values were slightly higher than the desired .08 cut-off, but this is not unexpected when sample size is small (*N* < 300), and can be ignored if other indices suggest good model fit [28]. Separating the feeling fat subscale into two separate factors (as per the pregnant cohort) yielded significant improvement in model fit (Δχ^2^ = 7.068, *p* < .01).

**Table 3:** Tests of measurement model fit for each subscale, non-pregnant cohort only (*n* = 148)

						**90% CI**	
**Subscale**	**χ**^**2**^	**df**	**CFI**	**SRMR**	**RMSEA**	**LOW**	**HIGH**
Feeling fat_1F	107.844	54	.936	.052	.082	.059	.105
Feeling fat_2F	90.776	53	.955	.047	.069	.044	.093
Attractiveness	3.252	5	1.000	.018	.000	.000	.091
Weight/shape salience	12.744	5	.969	.029	.102	.033	.174
Strength & fitness	17.778	9	.960	.044	.081	.019	.137

Notes: Feeling fat_1F and feeling fat_2F reflect uni-dimensional and two-dimensional representations of the feeling fat subscale items, respectively.

Comparisons between pregnant and non-pregnant women are shown in Tables 4, 5 and 6. In each instance, configural invariance (common number of factors) was established and formed a suitable baseline against which to test the increasingly stringent invariance assumptions. Regardless of which time point non-pregnant women were compared against, substantial declines in model fit (as evidenced by ΔCFI values) were observed for each of the subscales once factor loadings were constrained to equality across groups, with the following exceptions: (1) the attractiveness subscale (comparison with T1 pregnancy data), (2) strength and fitness subscale (comparison with T1), and (3) feeling fat - clothing specific (comparison with T2 and T3 data).

**Table 4:** Tests of invariance between non-pregnant and Time 1 pregnant data (at or after 16 weeks gestation)

							**90% CI**	
**Subscale**	**χ**^**2**^	**df**	**CFI**	**ΔCFI**	**SRMR**	**RMSEA**	**LOW**	**HIGH**
*Feeling fat_revf1*								
Configural	84.879	28	.954	-	.029	.079	.060	.099
Weak	108.850	34	.939	.015	.057	.083	.066	.100
Weak_rev	100.381	33	.945	.009^	.061	.080	.062	.098
Strong	218.299	41	.857	.088	.049	.116	.101	.131
*Feeling fat_revf2*								
Configural	77.405	10	.880	-	.014	.145	.116	.176
*Attractiveness*								
Configural	12.817	10	.991	-	.021	.030	.000	.071
Weak	16.726	14	.991	.000	.024	.025	.000	.062
Strong	25.461	19	.979	.012	.024	.032	.000	.062
*Weight/shape salience*								
Configural	46.348	10	.946	-	.034	.106	.076	.138
Weak	63.651	14	.926	.020	.052	.105	.080	.132
Weak_rev	50.322	13	.944	.002^	.044	.094	.068	.123
Strong	121.615	19	.846	.098	.080	.130	.108	.152
*Strength & fitness*								
Configural	56.429	18	.939	-	.051	.081	.058	.106
Weak	60.516	23	.940	.001	.053	.071	.050	.093
Strong	138.093	29	.826	.114	.055	.108	.090	.127

Notes: ^ compared against CFI for configural model; Feeling fat_revf1 and revf2 represent the feeling fat-general and feeling-fat clothing specific factors.

**Table 5:** Tests of invariance between non-pregnant and Time 2 pregnant data (at approximately 24 weeks gestation)

							**90% CI**	
**Subscale**	**χ**^**2**^	**df**	**CFI**	**ΔCFI**	**SRMR**	**RMSEA**	**LOW**	**HIGH**
*Feeling fat_revf1*								
Configural	48.101	28	.982	-	.028	.047	.023	.069
Weak	270.129	34	.787	.195	.048	.147	.131	.163
Weak_rev	58.731	33	.977	.005^	.028	.049	.028	.069
Strong	368.601	41	.704	.273	.035	.158	.143	.172
*Feeling fat_revf2*								
Configural	48.243	10	.933	-	.071	.109	.079	.141
Weak	51.004	14	.935	.002	.074	.091	.065	.118
Strong	88.185	19	.879	.056	.086	.106	.085	.129
*Attractiveness*								
Configural	8.245	10	1.000	-	.021	.000	.000	.052
Weak	107.336	14	.715	.285	.126	.144	.119	.170
Weak_rev	9.463	13	1.000	.000^	.021	.000	.000	.041
Strong	161.538	19	.565	.435	.022	.153	.131	.175
*Weight/shape salience*								
Configural	24.990	10	.965	-	.026	.068	.035	.102
Weak	174.262	14	.624	.341	.267	.189	.164	.214
Weak_rev	39.135	12	.956	.009^	.036	.084	.055	.114
Strong	489.143	19	.239	.717	.307	.277	.256	.299
*Strength & fitness*								
Configural	11.823	18	1.000	-	.028	.000	.000	.028
Weak	103.809	23	.780	.220	.174	.104	.085	.125
Weak_rev	13.623	21	1.000	.000^	.039	.000	.000	.023
Strong	381.024	29	.042	.958	.174	.194	.177	.212

Notes: ^ compared against CFI for configural model; Feeling fat_revf1 and revf2 represent the feeling fat-general and feeling-fat clothing specific factors.

**Table 6:** Tests of invariance between non-pregnant and Time 3 pregnant data (at approximately 32 weeks gestation)

							**90% CI**	
**Subscale**	**χ**^**2**^	**df**	**CFI**	**ΔCFI**	**SRMR**	**RMSEA**	**LOW**	**HIGH**
*Feeling fat_revf1*								
Configural	48.196	28	.983	-	.028	.047	.023	.069
Weak	319.830	34	.762	.221	.053	.162	.146	.178
Weak_rev	63.927	33	.974	.009^	.034	.054	.034	.074
Strong	440.803	41	.668	.306	.059	.174	.160	.189
*Feeling fat_revf2*								
Configural	52.649	10	.927	-	.072	.115	.086	.147
Weak	56.518	14	.928	.001	.076	.097	.071	.124
Strong	104.788	19	.854	.074	.095	.118	.097	.141
*Attractiveness*								
Configural	21.588	10	.963	-	.021	.060	.024	.095
Weak	86.296	14	.768	.195	.089	.127	.102	.153
Weak_rev	23.197	13	.967	.004^	.021	.049	.011	.081
Strong	137.085	19	.621	.346	.107	.139	.118	.161
*Weight/shape salience*								
Configural	28.940	10	.966	-	.034	.077	.045	.110
Weak	147.136	14	.763	.203	.262	.172	.147	.198
Weak_rev	32.047	12	.964	.002^	.034	.072	.042	.103
Strong	494.508	19	.152	.812	.032	.279	.258	.300
*Strength & fitness*								
Configural	52.276	18	.940	-	.050	.077	.053	.102
Weak	239.553	23	.624	.316	.165	.171	.152	.191
Weak_rev	62.135	21	.930	.010^	.052	.078	.056	.101
Strong	424.666	29	.313	.617	.146	.206	.189	.223

Notes: ^ compared against CFI for configural model; Feeling fat_revf1 and revf2 represent the feeling fat-general and feeling-fat clothing specific factors.

Based on modification indices provided for the poorly fitting weak invariance models, several factor loadings were freed from equality constraints in order to see whether partial invariance could be achieved. Freeing of these factor loadings across groups led to model fit that was comparable to the configural invariance model (see revised models in Tables 4, 5, 6, and 7 for the full list of the items that were freed from equality constraints). These revised weak invariance models were then compared against the strong invariance models in which item intercepts were also constrained to equality, and revealed substantial loss in model fit. Inspection of modification indices revealed a non-uniform pattern of cross-group differences in item intercepts (see Additional file 1). Given the absence of a clear pattern of response bias, the decision was made to terminate measurement invariance tests at this step rather than freeing more model parameters.

**Table 7:** Items which were freed from cross-group loading constraints

**Factor**	**Comparison groups**
*Feeling fat_revf1*	
Item 10: I hardly ever feel fat	Non-preg v T2 and T3 preg women
Item 28: I feel fat when I have my photo taken	Non-preg v T1 women
*Attractiveness*	
Item 3: People hardly ever find me sexually attractive	Non-preg v T2 and T3 preg women
*Weight salience*	
Item 11: There are more important things in life than the shape of my body	Non-preg v T2 and T3 preg women
Item 20: I hardly ever think about the shape of my body	Non-preg v T2 and T3 preg women
Item 32: I am preoccupied with the desire to be lighter	Non-preg v T1 women
*Strength*	
Item 16: I quickly get exhausted if I overdo it	Non-preg v T2 and T3 preg women
Item 43: I have never been strong	Non-preg v T2 and T3 preg women

Notes: Item numbers correspond with Ben-Tovim and Walker [19].

## Discussion

The present study addressed a significant gap in the women’s body image literature by evaluating the functioning of a commonly used measure of body image, the Body Attitude Questionnaire, BAQ [19], both across the phases of pregnancy and between pregnant and non-pregnant women. Collectively, our findings suggest that comparisons of body dissatisfaction between pregnant and non-pregnant women (at least based on the BAQ) are likely to be conflated by differential measurement biases that serve to undermine attempts to accurately assess potential differences in body dissatisfaction.

While adequate model fit was established for the subscales of BAQ when fit simultaneously for pregnant and non-pregnant women (i.e., configural invariance), subsequent cross-group equality constraints on factor loadings led in most instances to significant worsening of model fit and necessitated freeing the invariance assumption for several loadings in order to achieve acceptable fit. Poor fit for the subsequent, strong invariance model (with item intercepts set to equality across groups) revealed non-ignorable differences in the way pregnant and non-pregnant women responded to survey items. Measurement invariance tests were terminated at this step after inspection of modification indices revealed a non-uniform pattern of differences in item intercepts for the two groups – that is, in some instances, pregnant women gave more extreme responses than non-pregnant women, while in other instances non-pregnant women gave more extreme responses. A similar pattern of results was found when comparing across phases of pregnancy: configural and weak invariance assumptions held, but equality constraints on item intercepts led to significant worsening of model.

There are several possible explanations for non-invariance of item intercepts. Within the context of cross-group analyses, item intercept differences may be reflective of different response styles across groups [19,26]. That is, for the offending item(s), one group may have a greater tendency to provide extreme responses (higher or lower). While the present study is the first to demonstrate that body dissatisfaction measures do not function equivalently in pregnant populations, such findings are consistent with other research showing unanticipated differences in the way various populations respond to these measures. For instance, measurement non-invariance is often found across gender and culture, with males more likely than females to use extreme response profiles [29], and for individuals in eastern cultures to provide more middle response options (due to modesty) than western counterparts [30].

Within the context of within-subject designs (such as repeated assessment of pregnant women), it is more likely that non-invariant item intercepts reflect participants’ recalibration of the response scale [17]. That is, despite having the same level of dissatisfaction at T1 and T2, an individual may give a rating of ‘moderately agree’ at T1, but a rating of ‘slightly agree’ at T2 because her/his interpretation of what constitutes slight and moderate agreement change over time.

One common approach to deal with recalibrated response categories would be to collapse across blurred/non-distinct categories. For instance, non-invariance issues may be resolved if a 7 point response scale (1 = strongly disagree, 2 = moderately disagree, 3 = slightly disagree, 4 = neither agree nor disagree, 5 = slightly agree, 6 = moderately agree, and 7 = strongly agree) is changed to a 5 point scale by collapsing the slightly and moderately categories at each end of the scale (i.e., 1 = strongly disagree, 2 = moderately disagree, 3 = neither agree nor disagree, 4 = moderately agree, and 5 = strongly agree). The suitability of this approach depends on whether there are obvious categories to collapse or remove, a consideration that can be made by a knowledge expert. Further research is needed to evaluate whether changing the response options improves or diminishes the psychometric properties of the BAQ.

Differential response biases across groups can be dealt with by including covariates for response bias [31,32], provided the pattern of response bias is consistent across items. Unfortunately, this method could not be used for the present sample, as the pattern of item intercept non-invariance was complex, with no clear pattern of response bias.

### Limitations

It is worth noting the limitations of the present study. First, our two groups (pregnant and non-pregnant women) differed on the key demographics of age, BMI, exercise habits, relationship status, number of children, and history of mental illness. Some of these differences may be anticipated given the nature of the two groups. For instance, it is not unreasonable to expect that more pregnant women would be married or in a long-term relationship, to be older, have larger BMI, and/or have more children than non-pregnant women. While it is desirable – where possible – to match participants on these background variables, doing so would likely produce an atypical non-pregnant sample that does not represent the broader non-pregnant population.

Present findings also raise concerns about the dimensionality of the feeling fat subscale of the BAQ. Whereas the authors of this measure recommend a uni-dimensional model for these items, a two factor model was necessary to achieve acceptable model fit for the pregnant women in our sample. This two-factor model also produced significantly improved fit over the uni-dimensional model for our non-pregnant group. Therefore, some caution need be applied when interpreting invariance results for the feeling fat subcomponents as a two factor solution has not been previously reported in the literature. Despite replicating this two-factor solution across three stages of pregnancy, further examination of the dimensionality of the feeling fat items is warranted.

### Implications of present findings

These limitations notwithstanding, the present study shows the utility of measurement invariance tests for evaluating the suitability of a given self-report measure for cross-group and/or cross-temporal tests of group difference. Invariance tests revealed evidence of differential response styles for all subscales of the BAQ, and thus cast doubt on the accuracy of previous estimates of group differences (both between pregnant and non-pregnant women, and across the phases of pregnancy) which have relied on the BAQ [12,13].

In the absence of measurement invariance, researchers are unable to establish whether the level of body dissatisfaction experienced during pregnancy is commensurate with, or perhaps greater than, that reported pre-pregnancy. Nor are they able to determine whether peaks in an individual’s body dissatisfaction throughout pregnancy represent natural, benign fluctuations or whether they are potentially indicative of future mental health issues, such as post-natal depression. As a consequence, the BAQ as presently constituted is unable to provide clinicians and health care providers with information necessary to effectively monitor the well-being of pregnant women, in terms of attitudes toward their bodies during this life phase.

It is clear there is a need for a body dissatisfaction measure which functions appropriately in pregnant populations [13]. When creating and testing a suitable pool of items, researchers should be attentive to the presence of potential response biases (such as extreme response tendencies or acquiescence) in order to reduce the risk of non-invariant item intercepts as found in the present study [32]. Quantitatively-based decisions about which items to retain may also be supplemented with qualitative interviews of pregnant women to determine how response options may be recalibrated across the phases of pregnancy. Until such a measure has been devised and validated, it is recommended that body image researchers test for measurement invariance in their samples as a precautionary measure before drawing conclusions about group differences in body dissatisfaction which involve comparisons against groups of pregnant women.

## Conclusion

There is little doubt now that body image issues in obstetrics and gynecology impact negatively on women’s health and well-being [11]. Yet, alarmingly, a recent survey revealed that less than one third of physicians assessed for body image concerns during routine gynecologic and obstetric care [33]. This is surprising for two reasons: (1) obstetricians and gynecologists often act as primary care physicians for women over the life cycle, and (2) body dissatisfaction is often associated with negative psychological functioning, such as depression, and maladaptive behaviours, such as unhealthy eating and extreme weight loss behaviours. Screening for body dissatisfaction, extreme weight loss behaviours and /or a history of eating disorders, during routine obstetric and gynecological visits, should be considered by the physicians and other allied- health professionals who care for pregnant women [11]. However, this is not possible at present because a specifically designed measure for pregnancy has yet to be developed.

Given the non-uniform pattern of differences in item intercepts observed in the present study, it is unclear whether prior literature has under- or over-estimated the level of body dissatisfaction among pregnant women. Development of a psychometrically valid body image measure for use in pregnant populations would therefore help determine the direction and extent to which earlier estimates of differences in body dissatisfaction between pregnant and non-pregnant women [11], and also across the phases of pregnancy [12-16] were biased by measurement confounds identified in the present study, such as different response styles and potential recalibration of meaning of items across time points. Until this measurement issue has been resolved, available models and prescriptions regarding the fluidity and severity of body image concerns across pregnancy should be viewed with caution.

## Competing interests

The authors declare that they have no competing interests.

## Authors’ contributions

MFT participated in analysis and interpretation of results, and contributed to drafting of the manuscript. HS contributed to the design of the study, and drafting of the manuscript. BW and BH collected data for the study, conducted a literature search for the Background section and contributed to drafting of the manuscript. All authors have seen and approved the final version of the manuscript.

## Authors’ information

MFT is a Senior Lecturer in the School of Psychology, Deakin University, Melbourne, Australia. MFT teaches research methods in undergraduate and postgraduate courses. He is a member of the Centre for Mental Health and Well-being Research at Deakin University. MFT has a PhD in Psychology from Deakin University, and has published in the area of body image research.

HS is Associate Professor in the School of Psychology, Deakin University, Melbourne, Australia. HS has published extensively in the area of body image during pregnancy and post birth and also has expertise in the area of maternal and childhood obesity.

BW is a research assistant who has worked on this project.

BH is a PhD student on an NHMRC scholarship who is investigating psychosocial predictors of excessive gestational weight gain, including body image.

## Pre-publication history

The pre-publication history for this paper can be accessed here:

http://www.biomedcentral.com/1471-2393/12/91/prepub

## Supplementary Material

Additional file 1: Direction of differences in item intercepts for strong invariance models (modification indices >3.84) comparing pregnant v non-pregnant women. Click here for file
