# Supplementary material for: Body image during pregnancy: an evaluation of the suitability of the body attitudes questionnaire
Source: BMC Pregnancy Childbirth. 2012 Sep 6;12:91. doi: 10.1186/1471-2393-12-91 (PMC3500714; doi:10.1186/1471-2393-12-91)
Supplement: Additional file 1: — Direction of differences in item intercepts for strong invariance models (modification indices >3.84) comparing pregnant v non-pregnant women. [file 1471-2393-12-91-S1.doc]

Appendix A

Direction of differences in item intercepts for strong invariance models (modification indices >3.84) comparing pregnant v non-pregnant women

| Item | T1  (16 weeks gestation) | T2  (24 weeks gestation) | T3  (32 weeks gestation) |
| --- | --- | --- | --- |
| *Feeling fat-revf1* |  |  |  |
| Item 4: I get so worried about my shape that I feel I ought to diet | - | - | - |
| Item 8: I worry that other people can see rolls of fat around my waist and stomach |  |  | - |
| Item 10: I hardly ever feel fat |  | + |  |
| Item 28: I feel fat when I have my photo taken | + | + | + |
| *Attractiveness* |  |  |  |
| Item 3: People hardly ever find me sexually attractive |  | + | + |
| Item 40: People often compliment me on my looks |  | + | + |
| *Weight salience* |  |  |  |
| Item 11: There are more important things in life than the shape of my body |  | - | - |
| Item 20: I hardly ever think about the shape of my body |  | + | + |
| Item 32: I am preoccupied with the desire to be lighter | - |  |  |
| *Strength/fitness* |  |  |  |
| Item 16: I quickly get exhausted if I overdo it | - |  |  |
| Item 26: I have a strong body |  |  | + |
| Item 29: I try and keep fit | - |  | - |
| Item 37: I am bit of an Iron-Woman |  | - |  |
| Item 43: I have never been strong |  | + | + |

Notes: all comparisons relative to non-preg data; + indicates that the item intercept is higher for pregnant women; - indicates that the item intercept is lower for pregnant women. Item numbers correspond with Ben-Tovim and Walker [19].

Direction of differences in item intercepts for strong invariance models (modification indices >3.84) comparing pregnant women across time

| Item | T2  (24 weeks gestation) | T3  (32 weeks gestation) |
| --- | --- | --- |
| *Feeling fat-revf1* |  |  |
| Item 8: I worry that other people can see rolls of fat around my waist and stomach |  | - |
| Item 35: I often feel fat | - |  |
| *Feeling fat-revf2* |  |  |
| Item 5: I feel fat when I can’t get clothes over my hips | + | + |
| Item 14: I feel fat when I wear clothes that are tight around the waist | - | - |
| *Attractiveness* |  |  |
| Item 3: People hardly ever find me sexually attractive | - | - |
| Item 40: People often compliment me on my looks | + | + |
| *Weight salience* |  |  |
| Item 32: I am preoccupied with the desire to be lighter | + | + |
| *Strength/fitness* |  |  |
| Item 16: I quickly get exhausted if I overdo it |  | - |

Notes: all comparisons relative to T1 data (at or after 16 weeks gestation); + indicates that the item intercept is higher for pregnant women after T1; - indicates that the item intercept is lower for pregnant women after T1. Item numbers correspond with Ben-Tovim and Walker [19].
